# Impacts of the COVID-19 pandemic on access to healthcare among people with disabilities: evidence from six low- and middle-income countries

**DOI:** 10.1186/s12939-023-01989-1

**Published:** 2023-08-31

**Authors:** Xanthe Hunt, Shaffa Hameed, Shailaja Tetali, Luong Anh Ngoc, John Ganle, Lopita Huq, Tom Shakespeare, Tracey Smythe, Zeynep Ilkkursun, Hannah Kuper, Ceren Acarturk, Nanda Kishore Kannuri, Vu Quynh Mai, Rifat Shahpar Khan, Lena Morgon Banks

**Affiliations:** 1 Institute for Life Course Health Research, Department of Global Health, Faculty of Medicine and Health Sciences, Stellenbosch University, Room 4009, Education Building, Cape Town, South Africa; 2 International Centre for Evidence on Disability, London School of Hygiene and Tropical Medicine, London, UK; 3 Indian Institute of Public Health-Hyderabad, Hyderabad, India; 4 Center for Training and Research on Substance Abuse - HIV, Hanoi Medical University, Hanoi, Viet Nam; 5 School of Public Health, University of Ghana, Accra, Ghana; 6 BRAC Institute of Governance and Development, BRAC University, Dakar, Bangladesh; 7 Department of Psychology, Koc University, Istanbul, Türkiye; 8 Division of Physiotherapy, Department of Health and Rehabilitation Sciences, Faculty of Medicine and Health Sciences, Stellenbosch University, Cape Town, South Africa; 9 Center for Population Health Science, Hanoi University of Public Health, Hanoi, Viet Nam

**Keywords:** COVID-19, Pandemic, People with disabilities, Inclusive healthcare, Lockdown, Healthcare access

## Abstract

**Background:**

The pandemic has placed considerable strain on health systems, especially in low- and middle-income countries (LMICs), leading to reductions in the availability of routine health services. Emerging evidence suggests that people with disabilities have encountered marked challenges in accessing healthcare services and supports in the context of the pandemic. Further research is needed to explore specific barriers to accessing healthcare during the pandemic, and any strategies that promoted continued access to health services in LMICs where the vast majority of people with disabilities live.

**Methods:**

Qualitative in-depth interviews were conducted with persons with disabilities in Ghana, Zimbabwe, Viet Nam, Türkiye (Syrian refugees), Bangladesh, and India as part of a larger project exploring the experiences of people with disabilities during the COVID-19 pandemic and their inclusion in government response activities. Data were analysed using thematic analysis.

**Results:**

This research found that people with disabilities in six countries - representing a diverse geographic spread, with different health systems and COVID-19 responses - all experienced additional difficulties accessing healthcare during the pandemic. Key barriers to accessing healthcare during the pandemic included changes in availability of services due to systems restructuring, difficulty affording care due to the economic impacts of the pandemic, fear of contracting coronavirus, and a lack of human support to enable care-seeking.

**Conclusion:**

These barriers ultimately led to decreased utilisation of services which, in turn, negatively impacted their health and wellbeing. However, we also found that certain factors, including active and engaged Organisations of Persons with Disabilities (OPDs) and Non-Governmental Organizations (NGOs) played a role in reducing some of the impact of pandemic-related healthcare access barriers.

## Background

The COVID-19 pandemic and measures employed for its containment, such as lockdowns, have severely impacted people’s livelihoods [1–4], social support networks and access to health [5], education [6], and general wellbeing [7, 8] in ways which are only now beginning to be understood. Particularly salient have been impacts on access to healthcare services. The pandemic has placed considerable strain on health systems, especially in low- and middle-income countries (LMICs), leading to reductions in the availability of routine health services [9]. Moreover, people have become less willing to seek routine services, possibly out of fear of infection [10], and loss of household income has negatively impacted treatment seeking and adherence, and health [11, 12].

Equitable access to health is essential for the one billion people with disabilities globally [13, 14]. People with disabilities often have greater needs for healthcare: they require both the same general healthcare as people without disabilities (e.g., sexual and reproductive health, primary care), as well as healthcare needs associated with their impairments and underlying health conditions (e.g., rehabilitation or specific medications) [15–17]. Yet, people with disabilities are often underserved by existing health systems [18]. Even before the emergence of COVID-19, people with disabilities frequently encountered barriers to seeking and receiving quality, affordable healthcare services [19–23]. These challenges are due to a confluence of factors, including stigma [24], inaccessible services [19], lack of appropriate transport to and from healthcare facilities [19], and high costs [25, 26].

Emerging evidence suggests that people with disabilities have encountered marked challenges in accessing healthcare services and supports in the context of the pandemic [27–30]. For example, people with disabilities in the United Kingdom were much more likely to report difficulties accessing healthcare during the initial months of the pandemic compared to people without disabilities [29].

Further research is needed to explore specific barriers to accessing healthcare during the pandemic, and any strategies that promoted continued access to health services in LMICs where the vast majority of people with disabilities live [14, 31]. This evidence is essential to inform inclusive responses in future pandemics or share lessons learned and implement in similar settings. Consequently, this research set out to explore the experiences of people with disabilities in accessing healthcare during the COVID-19 pandemic between 2020 and 2021 in six LMICs.

## Methods

Qualitative in-depth interviews were conducted with persons with disabilities in Ghana, Zimbabwe, Viet Nam, Türkiye (Syrian refugees), Bangladesh, and India as part of a larger project exploring the experiences of people with disabilities during the COVID-19 pandemic and their inclusion in government response activities. For this paper, we are focusing on the experience of people with disabilities in accessing disability-related and general healthcare during the pandemic. Data were collected at different time points in 2020–2021 (Table 1).

### Selection and recruitment of participants

In each setting, interviews were conducted with between 20 and 60 people with disabilities. People with disabilities were recruited through different avenues in each setting. Modes of recruitment included:

- Previous quantitative surveys: in Bangladesh, people with disabilities were recruited from recently completed surveys run research team that included questions on disability. In Viet Nam, people with disabilities were recruited from the lists provided by the government of people certified as having disability.
- Organizations of Persons with Disabilities (OPDs)/Non-Governmental Organizations (NGOs): In all settings, at least a portion of participants were recruited through OPDs/NGOs working with people with disabilities, using membership/contact lists.

Participants were purposively selected using these techniques to maximise heterogeneity by gender, location, age (children, working age adults, older adults) and impairment types (vision, hearing, intellectual/cognitive, physical, psychosocial/mental). Participants were contacted by phone and invited to participate.

### Data collection

People with disabilities were interviewed relatively early in the pandemic (mostly between waves 1 and 2). In most settings, interviews were conducted remotely due to restrictions on in-person meetings at the time of data collection. In Ghana and Zimbabwe, national and local guidance allowed for at least a portion of interviews to be done in-person. Where interviews were conducted remotely, respondents were provided with mobile phone data to engage in the call.

All interviewers received a one to two-day training on study methods, disability, and research ethics. They also received in-depth feedback on all pilot interviews which were conducted. In-depth interviews were conducted using a semi-structured interview guide. The interviews lasted approximately an hour, and were audio and/ or video recorded, transcribed, and at least a portion translated into English. These guides covered similar domains but were adapted for each setting and piloted prior to use. The full interview guide explored the impact of COVID-19 amongst people with disabilities across multiple life areas (e.g., education, employment, social participation, household finances). The topic guide specifically asked people with disabilities to reflect on their experience accessing the healthcare they required both before the introduction of COVID-19 and at different stages of the pandemic. Participants were also asked to reflect on barriers and enablers to access, and important avenues for improving the inclusiveness of responses to future pandemics.

Details of country data collection are shown in Table 1.

**Table 1:** Details of data collection by site

Country	Regions	Data collection type	Date of data collection	Covid-19 restrictions in place over the recall period
Bangladesh	14 districts across 8 divisions of Bangladesh	Remote	April-August 2021	Nationwide lockdown, mandatory mask-wearing to receive services, border closures, police presence/fines enforce restrictions, closure of schools and non-essential businesses
Ghana	3 regions of Ghana: Northern, Ashanti, and Greater Accra	In-person and remote	May-July 2021	Mandatory mask-wearing (indoor and crowded outdoor spaces), closure of some non-essential businesses (e.g., clubs, cinemas), limits on large social gatherings
India	6 States of India: Tamil Nadu, Karnataka, Delhi, Telangana, Maharashtra, and Kerala	Remote	December 2020-March 2021	Movement restrictions, mandatory mask-wearing, school closures, restrictions on social gatherings.
Türkiye	Istanbul, Sultanbeyli	Remote	May-August 2021	Mandatory mask-wearing in public indoor and outdoor spaces, social distancing, travel restrictions, ban on mass gatherings, closure of some non-essential businesses (e.g., clubs, cinemas), school closures, nationwide weekend lockdowns (for periods of 2–3 months)
Viet Nam	Ha Noi, Da Nang, and Ho Chi Minh city	Remote	December 2021-March 2022	Mandatory mask-wearing in public indoor and outdoor spaces, travel restrictions, closure of some non-essential businesses (e.g., clubs, cinemas), restrictions on mass gatherings, school closures
Zimbabwe	Masvingo Province	In-person	May-June 2021	Mandatory mask-wearing (indoor and crowded outdoor spaces), closure of some non-essential businesses (e.g., clubs, cinemas), limits on large social gatherings

### Ethical considerations

Ethics approval for this study was obtained from the London School of Hygiene and Tropical Medicine and national review boards in each country: Medical Research Council of Zimbabwe (MRCZ) (No. MRCZ/A/2731) in Zimbabwe; Koc University Committee on Human Research (2020.306.IRB3.113) in Türkiye; Ethical Review Board For Biomedical Research of Hanoi University of Public Health (No. 427/2021/YTCC-HD3) in Viet Nam; Institutional Review Board, BRAC James P Grant School of Public Health, BRAC University (IRB Reference No. IRB-22 March’21 − 008) in Bangladesh); Institutional Ethics Committee, Indian Institute of Public Health Hyderabad (IIPHH/TRCIEC/22/3/2020); and Ghana Health Service Ethics Review Committee (GHS-ERC009/06/20) in Ghana. All research was conducted in accordance with the Declaration of Helsinki.

Local OPDs, NGOs, and other community networks known to the country research teams were asked to advertise the study. Lists of phone numbers of individuals interested in participating based on the initial advert were provided to study staff (usually the local Principal Investigator (PI) or project manager working under the PI’s supervision), who contacted them by phone to invite them to participate. They were then read the information sheet for the study by the researcher. Consent was obtained in a way which was accessible to the individual being interviewed. This meant that in some cases, simplified, easy read versions of information sheets were explained to participants, or the consent form was explained using sign language. If they were interested in participating, then an interview was scheduled for a time and platform (telephone, Skype, Teams, Zoom etc.) which suited them. Prior to the commencement of all interviews, informed consent was sought. Consent to participate was sought directly from all participants above the age of consent in each country. Consent from carers was also sought for children below the age of consent and for adults who lack capacity to consent on their own (e.g., people with severe intellectual/cognitive impairments). In these instances, researchers still sought input from participants directly where possible and relevant (e.g., children over the age of 10 years) and in these instances received assent from the participant. Both consent and assent were provided orally and recorded for remote interviews and written for in-person interviews.

Wherever possible, participants were interviewed directly. Adaptations were put in place to support the participation of people with different impairments. For example, sign language interpretation via video conference was available for people with profound hearing impairments. Simplified interview schedules were used for people with cognitive/intellectual impairments and for younger children. For some children and people with severe difficulties understanding or communicating with available adaptations (e.g., people who are Deaf, illiterate and with no knowledge of sign language; people with severe intellectual/cognitive impairments), interviews with caregivers or joint interviews with caregivers and the person with a disability were used. All data were stored in line with local internal review board (IRB) requirements, in password-protected formats and on secure devices. referral services were identified in the event that respondents reported concerns that were an immediate threat to their or others’ safety.

### Data analysis

Data analysis took place in several stages. Firstly, country specific analysis took place. Drawing on the local language transcripts, each country team conducted an in-depth thematic analysis of all transcripts and drafted a report of country-specific themes. This was done to establish a ‘true’ account of the findings based on original language analysis. Next, cross-country analysis took place. A proportion of transcripts (10–15 per country) were translated into English (using gold standard practices for translation, including forward and back translation) and independently coded by three researchers (XH, SH, LMB). This was done to allow for cross-cutting findings to be identified for the dataset as a whole (across countries). This team coded transcripts interpedently for a country at a time before meeting to discuss progress and resolve any issues arising. For this latter analysis, all transcripts were coded deductively in NVivo 12 (QRS International, 2022) using a coding framework developed using the semi-structured interview guide as a starting point. Additional codes were added based on findings emerging from the data. Themes were developed using thematic analysis. Similarities and differences between countries and sub-groups of participants (e.g., by gender, impairment type) were explored. Finally, the findings of the cross-country analysis were written up and compared to the findings from the country-specific analysis. Final themes and sub-themes for presentation in this paper were discussed with country teams through a group Zoom call to determine the final content.

## Results

Details of participants are outlined in Table 2. Themes and sub-themes identified through the thematic analysis are outlined in Table 3.

**Table 2:** Participant details^1^

Country	Number of participants	Age of participants	Gender distribution (%)	Impairment types (%)^2^
Bangladesh	60	< 19 years: 20 20–59 years: 31 > 60 years: 9	M: 50% F: 50%	Visual = 18.3% Hearing = 15% Physical = 16.6% Remembering & concentrating = 16.6% Self-care = 15% Communication = 11.6% Psychosocial = 2.4%
Ghana	58	< 17 years: 20 18–59 years: 38	M: 41% F: 59%	Visual = 26% Hearing and speaking = 26% Physical = 34% Mental/intellectual = 14%
India	61	< 18 years: 16 18–64 years: 32 > 65 years:13	M: 67% F: 33%	Physical: 24.5% Hearing: 13.11% Vision: 32.8% Intellectual/developmental: 29.5% Mental Health: 0%
Türkiye	60	< 18 years: 12 18–64 years: 40 > 65 years: 8	M: 55% F: 45%	Vision = 22% Hearing = 17% Physical = 52% Intellectual/developmental = 5% Communication = 5% Psychosocial = 8%
Viet Nam	23	18–40 years: 13 41–55 years: 6 > 56 years: 4	M: 53% F: 47%	Vision = 17% Hearing and speaking = 7% Physical = 43% Intellectual = 17% Psychosocial = 17%
Zimbabwe	24	< 20 years: 5 21–50 years: 10 > 50 years: 9	M: 46% F: 54%	Vision = 20% Hearing and speaking = 20% Physical = 33% Intellectual/behavioural = 16% Epilepsy = 16%

**Table 3:** Themes and sub-themes

Themes	Barriers to access	Impact of disrupted access to healthcare	Enablers of healthcare access to the pandemic
Subthemes	System restructuring	Poorer health and functioning	Local availability of services
	Costs and ability to pay	Feeling left behind	Telemedicine and home-based care
	Fear of illness	Decreased wellbeing	Role of OPDs/NGOs
	Lack of support		Increased social support

### Barriers to access

Most participants reported that they required regular access to health services related to their impairment and underlying health conditions (e.g., rehabilitation, medications, services for chronic conditions). Even before COVID-19, many reported difficulties to timely, affordable access to the services they required. However, the pandemic had created new or exacerbated existing barriers. A wide range of factors – including systems restructuring, costs, fear, and lack of support from public institutions – led to increased difficulties accessing healthcare among participants during the pandemic. Some of these challenges were direct impacts of pandemic itself, including fear of becoming ill or overstretched health systems. Curfew and other travel restrictions made it difficult to attend clinics. There were also barriers created by indirect impacts of the pandemic, such as job loss and increased financial strain.

#### System restructuring

In all settings, there were changes in health systems functioning during the pandemic, as health personnel and facilities were often reoriented to COVID-19 prevention and treatment activities. Consequently, many routine services were affected by staff and resource shortages. Many participants shared difficulties in accessing healthcare they had previously been receiving, pointing to difficulties getting appointment times. For example, a 40-year-old woman with a physical disability in Türkiye explained how “*…there aren’t many appointments. They began to rarely give appointments, only a small amount. Because a lot of pressure happened during COVID-19.”*

Further, in some settings, the closure of schools led to loss of linked health services that had previously been delivered in schools for children with disabilities. As one caregiver of a six-year-old boy with autism explained, her son used to receive occupational therapy, music, and speech therapy through his school. However, when schools closed, these services were lost. She explained that, as of the time of the interview, it had been two months without services.

#### Costs and ability to pay

Cost was a significant barrier to healthcare for people with disabilities during the pandemic. Although cost was a pre-pandemic barrier to seeking healthcare before the pandemic, the pandemic was reported to have affected prices and ability to pay.

Some participants felt that the cost of care had increased during the pandemic. Increased costs were, in some cases, linked to disruptions in services, as people were forced to seek alternative, often costlier sources for health services, particularly medications. Further, indirect costs associated with accessing healthcare were reported to have increased, particularly transport. When services that had previously been provided through the public sector were unavailable, some people sought private providers, if they could afford to, but those who could not pay for private care were excluded.

Participant: *I used to go to a clinic near my house to write me a medicine that I take periodically because I am sick with blood pressure.*

Interviewer: *What about the time of COVID-19?*

Participant: *It became difficult sometimes I could not buy it and I had to go and buy medicine directly from the pharmacy at a high price because the clinic was not available to give me medicine for free at the time of COVID-19.*

[58-year-old man with a physical disability from Türkiye]

In addition to increased prices, capacity to pay was also affected by economic downturns associated with the pandemic and restrictions. Many participants were working in the informal sector, without workplace protections, and in jobs that were heavily reliant on customer interactions. For instance, in Türkiye, where this study was conducted among a Syrian refugee population, difficulty receiving work permits saw participants over-represented in the informal sector and in precarious employment. As such, many individuals lost their livelihoods or experienced reduced income particularly during periods with the strictest restrictions (e.g., lockdowns, closures of non-essential businesses). As one participant noted:

*Yes, buying the medicine even during Covid was a major problem because getting money to buy the medicine was difficult but, in the past, I would use the money I got from selling my farming goods to buy it.*

[19-year-old man with multiple impairments from Zimbabwe]

#### Fear of illness

Many participants felt anxious attending healthcare facilities for fear of becoming infected with coronavirus. Hospitals in particular were seen as high risk. This concern was a particular problem for accessing disability-related healthcare, which was often delivered in large, tertiary institutions that were at the frontlines of the COVID-19 response. For example, a man in Viet Nam explained that he had not gone for rehabilitation services in almost two years due to fear of infection:

*Before the pandemic, I used to go to the hospital for the rehabilitation service to restore my hand and leg functions every two months… Nonetheless, I have not been to the hospital since the beginning of the pandemic because I’m frightened about the disease transmission. Extremely frightened!*

[50-year-old man with mental^3^ and physical disabilities in Viet Nam]

Public facilities were also seen as higher risk. Although more affordable to access, some participants noted that state-sponsored healthcare was often overcrowded and had inadequate hygiene. Private healthcare facilities were seen as safer. However, private facilities were not affordable for many.

#### Lack of support

Many participants reported requiring assistance to seek medical care. Many people with disabilities reported that, before the pandemic, when they went to healthcare services they often relied on members of the public, friends, family members, or healthcare workers to help them, by, for instance, providing mobility or communication support. However, given the restrictions on contact associated with the pandemic, these kinds of informal supports were more limited due to fear of infection or not wanting to be seen violating COVID-19 guidance. Further, some informal support structures were disrupted in other ways, such as by people moving to other areas in response to economic or other pressures brought about by the pandemic. These disruptions to informal support posed challenges to people with disabilities trying to get to healthcare facilities.

*You see, sometimes when I’m in need of something, I go to some friends to ask if they can be of help; but now that we are not able to visit people at will due to Covid.*

[29-year-old woman with a physical disability from Ghana]

### Impact of disrupted access to healthcare

The pandemic-related barriers to accessing healthcare discussed above resulted in negative impacts for many participants, including poorer health and functioning, and feeling left behind in government responses.

#### Poorer health and functioning

One of the most common impacts of pandemic-related barriers to accessing healthcare was that participants either discontinued or received less of the services and products they required. Some participants stopped taking their medications or reduced the dosage:

*I have stopped taking medicine since the pandemic’s beginning* [18 months]. *The most crucial need is food. So, I have to save the money to buy food. I can’t stand the hungriness but the painfulness of the conditions.*

[62-year-old man with a physical disability from Viet Nam]

*Now we don’t have a place to sell our stuff and get money for pills. We spend lots of time without pills, but we are supposed to always have pills.*

[53-year-old woman with epilepsy from Zimbabwe]

Others stopped going for regular check-ups and therapy sessions, including one man from Bangladesh who explained that he had stopped going for vision checks because he could no longer afford the hospital visits:

Participant: *They check how much vision (level of eyesight) is left in me. You see, my vision is reducing every moment. They just check how much is left. After checking, they write it down.*

Interviewer: *So, you didn’t go there due to the COVID-19, right?*

Participant: *No, no, I didn’t go there recently.*

Interviewer: *Do you have any plans to go there?*

Participant: *Well, at this moment, I don’t.*

[32-year-old man with a vision impairment from Bangladesh]

As a consequence of reduced healthcare access, many participants reported that their health and functioning was deteriorating. One caregiver from India reflected on her 6-year-old boy with cerebral palsy and noted how “*because of lack of physiotherapy his walking, his postures, they are not in that good shape right now.”* Similarly, other caregivers worried that disruptions to healthcare were affecting their child’s development and resulting in the loss of previously attained milestones. Another participant described her and her husband’s worsened health and functioning:

Participant: *In the last two years, “cô vít cô veo” [COVID-19 pandemic] has prevented us [the participant and her husband who also live with a physical disability] from going to a hospital. Even its hurt to die, we dare not to go to a hospital. Well, and now we’re like “lá mùa thu” [autumn leaves which are yellow and almost shed], maybe gone soon. My groin joint is more painful four or five times than before [the COVID-19 pandemic] because I can’t go to a hospital and can’t walk around. My husband’s arms and legs are weaker and weaker.*

[59-year-old woman with a physical disability from Viet Nam]

#### Feeling left behind

Exclusions and challenges in accessing services by people with disabilities during the pandemic had the effect of making people feel that they had been left behind. Some participants felt that while services commonly used by people without disabilities had been maintained during the pandemic, auxiliary services, including the therapies on which they rely to function optimally, had fallen to the wayside. One participant articulated this in terms of the government de-prioritising non-emergency services:

*[Physiotherapy and similar therapies] are very badly affected and government I think could not, but it was maybe not intentionally, not yet something that was overlooked but I think the times were such compelling and pressing that the government could not also look at that.*

[30-year-old man with a vision impairment from India]

Others reported that they felt there had been a widespread disregard for people with disabilities needs in the design and delivery of COVID-19 containment measures and the COVID-19 response more broadly. As one respondent concluded:

*…there was no measure/arrangement for persons with disabilities.*

[49-year-old man with a physical disability from Ghana]

### Enablers of healthcare access to the pandemic

#### Local availability of services

In general, there was a greater continuity of care for services delivered locally – for instance, where medications were dispensed from local pharmacies rather than hospitals. In one setting, doctors did home visits to people’s houses.

Interviewer: *But is the doctor regularly available during the COVID-19 as well?*

Participant: *Yes.*

Interviewer: *Does he come here regularly even during the COVID-19?*

Participant: *The doctor is available here all the time.*

Interviewer: *Okay. So, you didn’t face any trouble even in the middle of the COVID-19 (pandemic), right?*

Participant: *Right.*

[55-year-old man with multiple impairments from Bangladesh]

#### Telemedicine and home-based care

A few participants, mainly in Bangladesh and India, reported the use of telemedicine to overcome access barriers to in-person services. The main users were children with disabilities who had been previously receiving services in schools and were connected to online support by their school (although not all children with school-based services were connected to online health services). For example, a caregiver noted that her son was receiving online physiotherapy, where a therapist would provide a demonstration of what they should do with him in the week that followed. However, she noted that the physiotherapy time slots available clashed with his schooling and that she did not have the equipment or training to deliver services of the same quality as a professional.

Participant: *Like we used to go to vision therapy weekly five days and then again physio for three days, and occupational two days. So, it has completely moved to online now and, the timing is also not [great]… because school is also going online…And the things the therapist has, like some toys, or some things which is required to do a therapy, that is not present at our home. All the things, we can’t get, Ok? For there is somewhat compromise in the quality. Ok?*

[Caregiver of an 8-year-old boy with cerebral palsy from India]

Still, telemedicine and home-based care were not widely used in any setting. Even when available, other barriers prevented uptake. For example, in Bangladesh, barriers to access included low awareness of the availability of services as well as lack of access to required technology (e.g., internet/data, computer/smartphone).

#### Role of OPDs/NGOs

Many participants had ties to OPDs and NGOs working on disability due to the means of recruitment. Participants frequently noted that OPDs and NGOs had been helpful to them during the pandemic, for example providing financial support or food packages. There were also accounts, from participants, of receiving special allowances due to their medical needs, allowances which could allow them to meet healthcare costs. For instance, the Turkish Red Crescent runs a programme for refugees called Kizilaykart which is a cash-based humanitarian relief scheme which participants described receiving assistance from:

Participant: *Red crescent card, yes. We have the red crescent card…*.

Interviewer: *And did this change in some way, Aunt? Increase or decrease?*

Participant: *Yes, it increased. They increased it. So, we used to get 125, then it became 155 and now for the situations – for medical cases. Like now my difficult health situation, yes now it is 250 per person.*

[52-year-old woman with a hearing impairment from Türkiye]

In other settings, participants described OPDs providing food packages. Participants also noted that OPDs were well-placed to identify and distribute support to people with disabilities and that they often filled roles that governments had not. For example, a 56-year-old man with a vision impairment in Ghana noted that OPDs were important implementers “*because the Blind they know where the Blind people are, and the physical knows where they are, and it comes.”*

## Discussion

This research found that people with disabilities in six countries - representing a diverse geographic spread, with different health systems and COVID-19 responses - all experienced additional difficulties accessing healthcare during the pandemic. Key barriers to accessing healthcare during the pandemic included changes in availability of services due to systems restructuring, difficulty affording care due to the economic impacts of the pandemic, fear of contracting coronavirus, and a lack of human support to enable care-seeking. These barriers ultimately led to decreased utilisation of services which, in turn, negatively impacted their health and wellbeing. However, we also found that certain factors, including active and engaged OPDs/NGOs played a role in reducing some of the impact of pandemic-related healthcare access barriers.

Concerns over disruptions in accessing healthcare amongst people with disabilities were raised widely at the start of the pandemic [27, 32, 33]. This research and other studies highlight that these concerns were well-founded. For children, for example, school closures led to the loss of essential therapeutic and rehabilitation services delivered through schools in many countries including the UK [34], Italy [35], and India [36]. Caregivers have reported a very negative impact of the loss of services and supports, and many have felt ‘left on their own to meet complex educational, sensory-related, medical and social care needs’ of young people with disabilities [34]. A French survey among 1000 caregivers of children with disabilities found that the pandemic had led to drastic reductions in children’s engagement in healthcare and therapies [37]. In the US, 80% of children with behavioural and mental health needs rely on school-based services for therapy [38]. As such, school closures have meant a far-reaching loss of critical health resources for children with disabilities. In order for health disparities between children with and without disabilities not to worsen, it is imperative that public health planning in the future takes these circumstances into account and recognise the role continuity of education and services delivered through schools play in continuity of care for young people with disabilities. For adults, disruptions were similarly damaging. A recent review by McBride-Henry [39] and colleagues notes that people with disabilities struggled to access healthcare services during the pandemic, resulting in feelings of ‘invisibility’ and poor mental health. These authors also highlight that people with disabilities with compounding vulnerabilities (such as ethnic minorities, or those of low socioeconomic status) were at the most risk of experiencing disruptions in healthcare access [39].

In the present work, rising costs and reduced ability to pay were also factors affecting healthcare access, which was found in some other studies, including in Japan [40]. Affordability of healthcare for people with disabilities has been a widespread challenge even before COVID-19. However, COVID-19 and the emerging cost of living crisis have now exacerbated these challenges. People with and without disabilities were affected by job and earnings losses during the pandemic, however people with disabilities appear disproportionately affected. For example, people with disabilities are overrepresented in informal employment [41], which is often not covered by employment protections and is heavily reliant on customer interactions that were restricted during periods of lockdowns and business closures. In a survey of the three largest cities in Viet Nam, people with disabilities were three times more likely to report having lost their job during the pandemic and 20% more likely to report their household income had decreased compared to people without disabilities [42]. People with disabilities also already faced a heightened risk of poverty [43], meaning many had less of a safety net to withstand short-term shocks such as the income loss during restrictions or rising costs due to inflation. There is also some evidence that inflation has disproportionately affected healthcare, including medications and assistive devices [44, 45]. For example, in Türkiye, inflation was 80.21% in August 2022, and one of the most affected costs were medicine [46]. This fits a global pattern: in the Maldives, for instance, 8% for assistive products and 9% for medicines in the first quarter of 2022, compared to an overall national inflation rate of 0.6% [47].

Fear of infection was also a barrier to seeking care, particularly in settings that had high COVID-19 caseloads. This fear appears well-founded for many, given evidence that some people with disabilities overall are at higher risk of COVID-19 infection and of negative health outcomes, including death [48–50]. Older adults, people with learning disabilities, and people with certain underlying chronic conditions are at particularly heightened risk.

Finally, disruptions to informal support also impacted health and access to health services. Personal assistance and human support are often delivered through informal networks of family or friends or community members, particularly in settings that lack formal social care services. These informal care networks appeared disrupted during the pandemic, due to regulations on social distancing and universal difficulties experienced by many members of the community. As early as March 2020, commentators were already warning of the possible implications which measures such as physical distancing or self-isolation might have on the provision of human support for people with disabilities who rely on assistance for medical and personal care [51]. These authors and others [52, 53] called for mitigation strategies to allow care workers and family members to continue to safely support people with disabilities. However, this study indicates that gaps still remained in the provision of personal assistance, which had wide ranging impacts on people with disabilities’ health, participation, and well-being.

Our study also revealed that the cumulative effect of these barriers meant that people stayed out of care, and that their well-being and health deteriorated during the pandemic. People with disabilities reported worsening health during the pandemic resulting from loss of disability-related and routine healthcare secondary to increased financial strain and pandemic-related barriers to accessing services. It also reinforced perceptions that the needs and concerns of people with disabilities were not adequately considered by governments. This perception is in line with findings from a recent scoping review of key learning points emerging from the COVID-19 literature [54], which found that public-health policies and strategies during the pandemic have often been made without an awareness and involvement of people with disabilities, their family, or carers.

It is also important to note that participants highlighted that certain factor played a role in minimising the impact of pandemic-related healthcare access barriers. These included locally available and accessible services, telemedicine interventions and home-based care, social support, and the role of OPDs/NGOs in supporting people with disabilities. Whilst informal social networks were often disrupted and the vast majority of participants experienced isolation, those who had increased social support found that the community support helped to maintain their resilience, wellbeing, and access to services. Additionally, local service delivery appeared protective of people with disabilities’ health. While participants expressed fear of seeking services at crowded public facilities, they also noted the relative ease with which they could adhere to medication regimens and treatment protocols when they were locally available outside of hospital settings, including through community-based pharmacies or clinics, or through mobile healthcare workers. Telemedicine was not widely used by study participants; however, other studies have shown that it holds promise in improving access to rehabilitation and other health services for people with disabilities even after it is no longer a pandemic-related necessity [55–57]. Complementary interventions - and evaluations of these interventions - are needed to support widespread uptake of telemedicine, such as by providing people with disabilities with required technology and training to use it.

Our study feeds into the emerging evidence on the positive role played by OPDs in the pandemic, including in advocating for a more inclusive policy response from governments, and in providing practical support to people with disabilities [58]. Indeed, advocacy briefs, policy notes, and other documentation released at various stages during the pandemic by civil society organisations have noted the significant role played by OPDs in terms of planning, programming, and advocating for people with disabilities in the context of the pandemic and possibly exclusionary governmental responses to it [59–61]. A rapid assessment of the effects of the COVID-19 pandemic on OPDs in Bangladesh, Nigeria and Zimbabwe reported that OPDs played a critical role responding to gaps in provision of key services due to non-inclusive planning [60]. However, the same study noted that OPDs experienced significant reductions to funding and operational capacity during the pandemic. These constraints, coupled with the fact that provision of healthcare services is often outside of the purview of many OPDs’ standard operations, meant that the role played by OPDs in bridging the gap between need to healthcare, and services, while important, was somewhat limited. However, the role which these organisations can play in linking people with disabilities to services, advocating for inclusion, and providing other forms of assistance to people with disabilities to enable healthcare-seeking, should be capitalised on and further invested in for emergency response planning going forward. This said, the reach of OPDs can be limited in some settings: for example, in a study that included survey data from nine countries, women, older adults, people with intellectual and communication impairments and people living in poverty and in rural settings were less likely to be affiliated with an OPD [62]. As such, alternative ways to enable access to care for people with disabilities are needed, to ensure that the most marginalised are reached during crises.

### Limitations

This study has several limitations that should be considered when interpreting the results. The first is that participants were mainly recruited through OPDs. OPDs, while extremely valuable networks for people with disabilities, are not necessarily representative of all people with disabilities in a setting [63], and so our sample may be biased in systematic ways which reflect the differences between people who are, and are not, part of OPD networks. It is also worth reflecting on how remote data collection impacted on the study. In most settings, remote data collection was used, meaning that people with disabilities were interviewed via telephone or videoconference. This meant that interviewers did not have access to some of the non-verbal cues which form an important part of interpersonal communication in in-person interactions, and this may have limited the depth of participants’ accounts in some instances. Further, relying on these forms of communication excluded people without access to the required technology. This is an important limitation as in some of the sites, rates of cell phone and computer access were low. Next, while accommodations were made as extensively as possible to ensure that people with disabilities were able to engage in the research, remote interviewing was particularly difficult for people with intellectual disabilities and communication impairments to utilise. This may have resulted in interviews with people with specific impairment types lacking depth compared to interviews with others. Finally, participants from Türkiye were all Syrian refugees for whom there are many NGOs providing targeted services. As such, findings should not be generalized the host community in Türkiye.

## Conclusion and directions for future research

In most countries, there are no longer wide-ranging restrictions such as there were during the start of the pandemic. However, the aftershocks of the pandemic are still being felt, including in a burgeoning cost of living crisis [64]. Findings from studies such as those presented in this paper foreground a need for more research on strategies which allow people with disabilities to maintain access to needed services in the event of shocks, including future pandemics, climate, or humanitarian crises.

In the wake of crises, there is often a call to ‘build back better’ - to learn from mistakes made and utilise the opportunities of a recovering healthcare system to improve services moving forward. The experiences of adults and children with disabilities in the context of COVID-19 were marked by a range of unintentional exclusions and profound challenges accessing health services. If this clarion call to move forward with better, stronger, more responsive healthcare systems is to be answered, disability inclusion must be centred. Further research is required to trial interventions that can support access of people with disabilities to required health services, including during times of crisis.

## Data Availability

The datasets used and/or analysed during the current study are available from the corresponding author on reasonable request.

## Footnotes

1 Data on demographic information were collected using different forms in each country, and so some categories of reporting cannot be standardised

2 For impairment type, % can be more than 100% (i.e., people with multiple disabilities were counted in each category, so if a person had a physical and hearing impairment, they were in both the physical and hearing categories).

3 In Viet Nam, impairment type data were validated against participants’ certificate of disability. In Viet Nam, the term mental disability is used to cover psychiatric and psychosocial impairments (and can be distinguished from intellectual disabilities).
